# A real-world study of ublituximab based on the WHO-VigiAccess and U.S. food and drug administration’s adverse event reporting system databases

**DOI:** 10.3389/fimmu.2026.1738185

**Published:** 2026-02-13

**Authors:** Jiagnang Cao, Qinguo Shen, Yan Zou

**Affiliations:** 1 Institute of Clinical Pharmacy Research, The Affiliated Nanhua Hospital, Hengyang Medical School, University of South China, Hengyang, Hunan, China; 2 State Key Laboratory of Integration and Innovation of Classic Formula and Modern Chinese Medicine, Linyi, Shangdong, China

**Keywords:** adverse events, food and drug administration adverse event reporting system, multiple sclerosis, ublituximab, WHO-VigiAccess

## Abstract

**Objective:**

The study bridge statistical pharmacovigilance signals to clinically actionable insights by systematically characterizing adverse event (AE) patterns associated with ublituximab and identifying previously unrecognized risks. We aim to establish a quantitative framework to inform real-world safety monitoring protocols and regulatory risk-benefit evaluations.

**Methods:**

We systematically analyzed AE reports associated with ublituximab through a comprehensive retrospective study of FAERS and VigiAccess databases (data cutoff: December 29, 2024). Utilizing five validated disproportionality analysis methods—the Reporting Odds Ratio (ROR), Proportional Reporting Ratio (PRR), Bayesian Confidence Propagation Neural Network (BCPNN), Medicines and Healthcare Products Regulatory Agency algorithm (MHRA), and Multi-Item Gamma Poisson Shrinker (MGPS).

**Results:**

Our investigation integrated 2, 245 case reports from the FAERS with 2, 037 entries in VigiAccess database. Signal detection via the ROR and PRR uncovered 52 preferred term (PT)-level signals significantly associated with ublituximab in the FAERS database, corresponding to 20 system organ class (SOCs). Notably, four SOCs - encompassing cardiovascular disorders, vascular pathologies, urinary system conditions, and social environment factors - were identified with disproportionality signals but currently lack documentation in approved prescribing information. We identified 8 frequently reported PTs (hypoaesthesia, fall, gait disturbance, paraesthesia, balance disorder, alopecia, tremor, and somnolence) within the top 50 AEs showed disproportionality signals but remain unlisted in the current drug lable.

**Conclusion:**

This study has identified confirmed positive signals associated with ublituximab and revealed potential signals of interest. These findings highlight the need for further regulatory review to assess whether the identified signals justify updates to drug labeling and therapeutic guidelines. Future investigations should build upon the AE signals established in this research, with further investigations warranted to elucidate both the clinical incidence rates of these AEs and their definitive causal relationships with ublituximab.

## 1 Introduction

Multiple Sclerosis (MS) represents a chronic autoimmune disorder of the central nervous system (CNS), characterized by a persistent interplay of inflammatory processes, demyelination, and axonal degeneration (1). MS demonstrates a global distribution, with its prevalence exhibiting a progressive upward trend (2). Patients with MS typically experience disease onset in their 30s, with progressive disability accumulation emerging in their 50s or 60s (3). Approximately 80% of them experience pain, and 50% suffer from paralysis. This disease is classified into three distinct types or stages: Relapsing-Remitting MS, Secondary Progressive MS, and Primary Progressive MS (4). The relapsing-remitting pattern is one of the hallmark characteristics of this disease. This complex pathophysiological triad reflects the intricate nature of MS and underscores the pressing need for targeted therapeutic strategies to curb these harmful processes.

Ublituximab (brand name BRIUMVI), developed by TG Therapeutics, is a glycoengineered anti-CD20 monoclonal antibody designed for the treatment of MS (5). B cells have been established as key players in the pathogenesis of MS (6). As the first anti-CD20 antibody for MS treatment, ublituximab primarily depletes B cells through enhanced antibody-dependent cellular cytotoxicity (7). Like the other three anti-CD20 monoclonal antibodies currently used for MS treatment—rituximab, ocrelizumab, and ofatumumab—ublituximab depletes B cells while sparing long-lived plasma cells (8). In the phase 3 ULTIMATE I and II trials, ublituximab showed significant benefits in lowering the annualized relapse rate in MS patients, as well as reducing the total number of gadolinium-enhancing (Gd^+^) T1 lesions and new or enlarging T2 lesions (9). However, as the clinical use of this drug expands, the current safety data are limited to clinical trials, restricted by sample size and follow-up duration, leaving the real-world risk profile of ublituximab incomplete.

The FDA Adverse Event Reporting System (FAERS), centered on the United States, reflects active surveillance and reporting preferences in clinical practice (10), while VigiAccess integrates global spontaneous reporting data, encompassing a broader population (11). Both databases draw on extensive data sources, including healthcare professionals, consumers, and pharmaceutical companies. Through complementary analysis of FAERS and VigiAccess, we enhance signal detection sensitivity, identify potential AE signals, and provide scientific evidence to inform clinical practice and drug regulation.

This study aims to extract data on ublituximab-related reports from the FAERS and VigiAccess databases, quantify the strength of adverse event (AE) signals, and identify novel AE signals potentially undetected in prior research or clinical trials, thereby providing a comprehensive safety assessment of ublituximab. Stratified analyses across age groups and genders lay the foundation for precisely identifying AE risks in specific populations. The publicly accessible data mining results not only enhance public awareness of the safety profile of ublituximab, empowering patients to better understand and manage their treatment, but also support clinicians in making informed therapeutic decisions. Furthermore, these findings provide scientific evidence for drug regulatory agencies’ pharmacovigilance efforts and offer new directions for drug development, potentially guiding improvements in drug design to minimize AEs.

## 2 Materials and methods

### 2.1 Data sources

**Figure 1:**
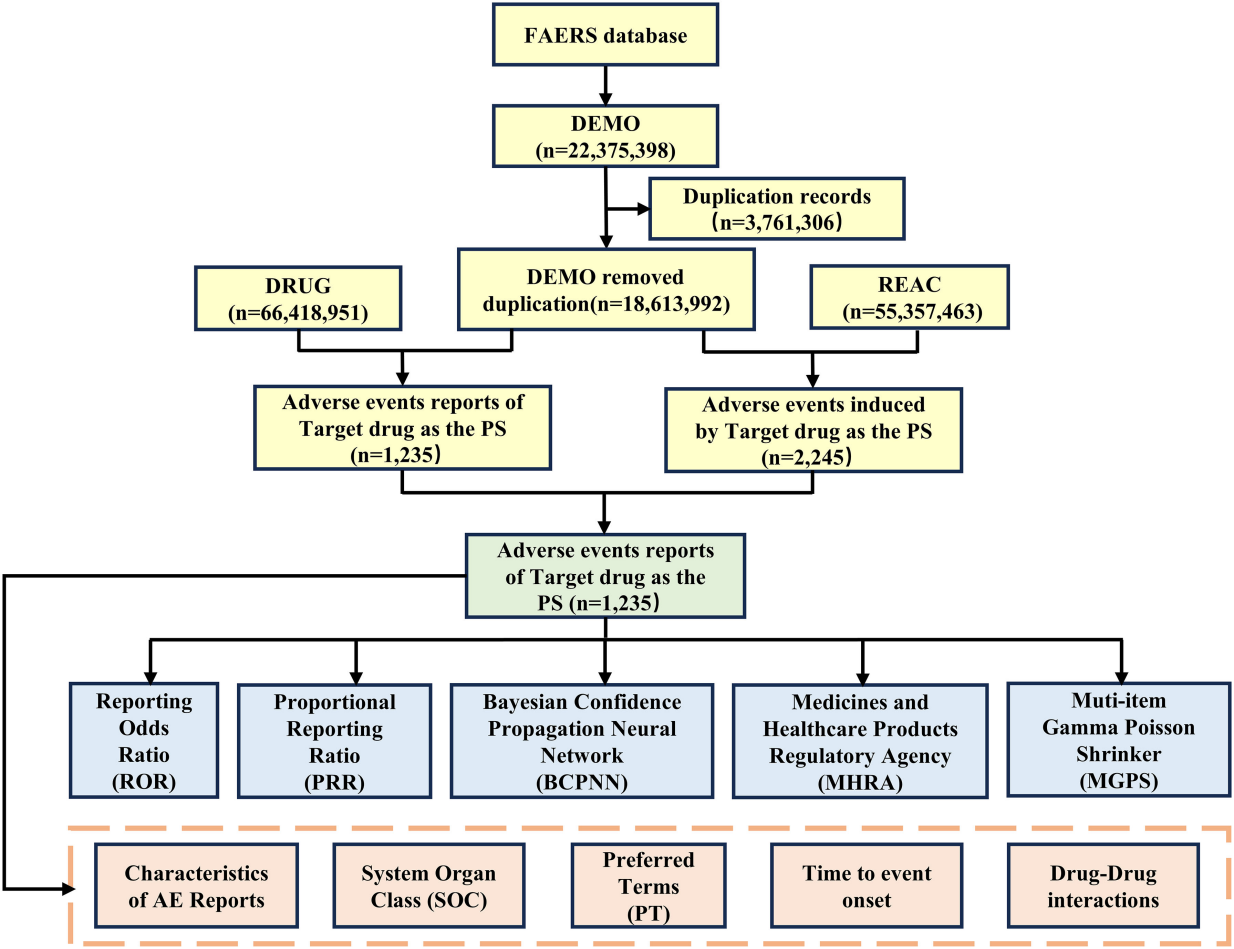
The flow diagram of selecting AEs of ublituximab in the overall population from FAERS database.

The FAERS and VigiAccess databases provide invaluable resources for monitoring drug AEs. We collected all reports from both databases (data cutoff: December 29, 2024). After deduplication of raw data, AE reports were stratified by drug and reaction associations. Pharmacovigilance algorithms—including the Reporting Odds Ratio (ROR), Proportional Reporting Ratio (PRR), Bayesian Confidence Propagation Neural Network (BCPNN), Medicines and Healthcare Products Regulatory Agency algorithm (MHRA), and Multi-Item Gamma Poisson Shrinker (MGPS)—were applied to identify signals, complemented by analyses of system organ class (SOC) and preferred term (PT) classifications, temporal patterns, and drug interactions. The granularity and hierarchical structure of the Medical Dictionary for Regulatory Activities (MedDRA) enable accurate AE coding for analysis (12). AEs were classified according to the latest version of the MedDRA 27.1. **Figure 1** illustrates the detailed data screening process, using FAERS as an example.

### 2.2 AE signal detection and statistical analysis methodology

This study employed a retrospective quantitative analysis approach to investigate the characteristics of AEs associated with ublituximab in real-world settings by analyzing all AE reports from the FAERS and VigiAccess databases. In FAERS, the methods used to assess the association between ublituximab and AEs include the Reporting Odds Ratio (ROR), Proportional Reporting Ratio (PRR), Bayesian Confidence Propagation Neural Network (BCPNN), Medicines and Healthcare Products Regulatory Agency algorithm (MHRA), and Multi-Item Gamma Poisson Shrinker (MGPS) (13). The corresponding calculation formulas are shown in **Table 1**.

**Table 1:** Methods, formulas, and thresholds of ROR, PRR, BCPNN, and EBGM.

Method	Formula	Threshold
ROR	$\text{ROR}=\frac{\left(\text{a}/\text{c}\right)}{\left(\text{b}/\text{d}\right)}=\frac{\text{ad}}{\text{bc}}$	a≥3 and 95% CI (lower limit)>1
	$\text{SE}(\ln\text{ROR})=\sqrt{\left(\frac{1}{\text{a}}+\frac{1}{\text{b}}+\frac{1}{\text{c}}+\frac{1}{\text{d}}\right)}$	
	$95\text{\% CI}={\text{e}}^{\ln(\text{ROR})\pm 1.96\sqrt{\left(\frac{1}{\text{a}}+\frac{1}{\text{b}}+\frac{1}{\text{c}}+\frac{1}{\text{d}}\right)}}$	
PRR	$\text{PRR}=\frac{\text{a}/(\text{a}+\text{b})}{\text{c}/(\text{c}+\text{d})}$	a≥3 and 95% CI (lower limit)>1
	$\text{SE}(\ln\text{PRR})=\sqrt{\left(\frac{1}{\text{a}}-\frac{1}{\text{a}+\text{b}}+\frac{1}{\text{c}}-\frac{1}{\text{c}+\text{d}}\right)}$	
	$95\text{\% CI}={\text{e}}^{\ln(\text{PRR})\pm 1.96\sqrt{\left(\frac{1}{\text{a}}-\frac{1}{\text{a}+\text{b}}+\frac{1}{\text{c}}-\frac{1}{\text{c}+\text{d}}\right)}}$	
	${\text{x}}^{2}=\frac{{\left(ad-bc\right)}^{2}(a+b+c+d)}{\left(a+b)(a+c)(c+d)(b+d\right)}$	
BCPNN	$\text{IC}=\log_{2}\frac{\text{p}(\text{x},\text{y})}{\text{p}(\text{x})\text{p}(\text{y})}=\log_{2}\frac{a(a+b+c+d)}{\left(a+b)(a+c\right)}$	IC025>0
	$\text{E}(\text{I}C)=\log_{2}\frac{\left(a+\gamma 11)(a+b+c+d+\alpha )(a+b+c+d+\beta \right)}{\left(a+b+c+d+\gamma )(a+b+\alpha 1)(a+c+\beta 1\right)}$	
	$\text{V}(\text{IC})=\frac{1}{{\left(\ln2\right)}^{2}}\{[\frac{(\text{a}+\text{b}+\text{c}+\text{d})-\text{a}+\text{γ}-\text{γ}11}{\left(\text{a}+\text{γ}11)(1+\text{a}+\text{b}+\text{c}+\text{d}+\text{γ}\right)}]+[\frac{(\text{a}+\text{b}+\text{c}+\text{d})-(\text{a}+\text{b})+\text{α}-\text{α}1}{\left(\text{a}+\text{b}+\text{α}1)(1+\text{a}+\text{b}+\text{c}+\text{d}+\text{α}\right)}]+[\frac{(\text{a}+\text{b}+\text{c}+\text{d})-(a+c)+\beta -\beta 1}{\left(\text{a}+\text{c}+\text{β}1)(1+\text{a}+\text{b}+\text{c}+\text{d}+\text{β}\right)}]\}$	
	$\gamma =\gamma 11\frac{\left(\text{a}+\text{b}+\text{c}+\text{d}+\text{α})(a+b+c+d+\beta \right)}{\left(a+b+\alpha 1)(a+c+\beta 1\right)}$	
	$\text{I}C-2SD=E(IC)-2\sqrt{V(IC)}$	
EBGM	$\text{EBGM}=\frac{a\times (a+b+c+d)}{\left(a+c)(a+b\right)}$	EBGM05>2
	$95\text{\% CI}={\text{e}}^{\ln(\text{EBGM})\pm 1.96\sqrt{\left(\frac{1}{\text{a}}+\frac{1}{\text{b}}+\frac{1}{\text{c}}+\frac{1}{\text{d}}\right)}}$	

These calculations are based on a fourfold table for disproportionality analysis (**Table 2**). A represents the number of cases of a specific AE attributed to the target drug; b denotes the number of cases of other AEs associated with the target drug; excluding the specific AE. c signifies the number of cases of the specific AE caused by drugs other than the target drug; d indicates the number of cases of other AEs caused by drugs other than the target drug, excluding the specific AE. AEs unrelated to ublituximab were thoroughly excluded from the analysis using the aforementioned methods. Statistical analysis and visualization were conducted using Python, SAS 9.4, and Microsoft Office Excel 2021.

**Table 2:** Two-by-two contingency table for disproportionality analysis.

Item	Target AEs reported	Other AEs reported	Total
Target drugs	a	b	a + b
Other drugs	c	d	c + d
Total	a + c	b + d	a + b + c + d

## 3 Results

### 3.1 Basic information on the AE reports

As of December 29, 2024, the FAERS database and VigiAccess database contained 1, 235 and 1, 042 AE reports for ublituximab, respectively, with 2, 245 and 2, 037 AEs recorded in AE reports. FAERS had more patients and reports than VigiAccess. The first ublituximab - related AE was documented in VigiAccess in 2015. In FAERS, 183 (18.3%) reports were serious reports.

**Figure 2:**
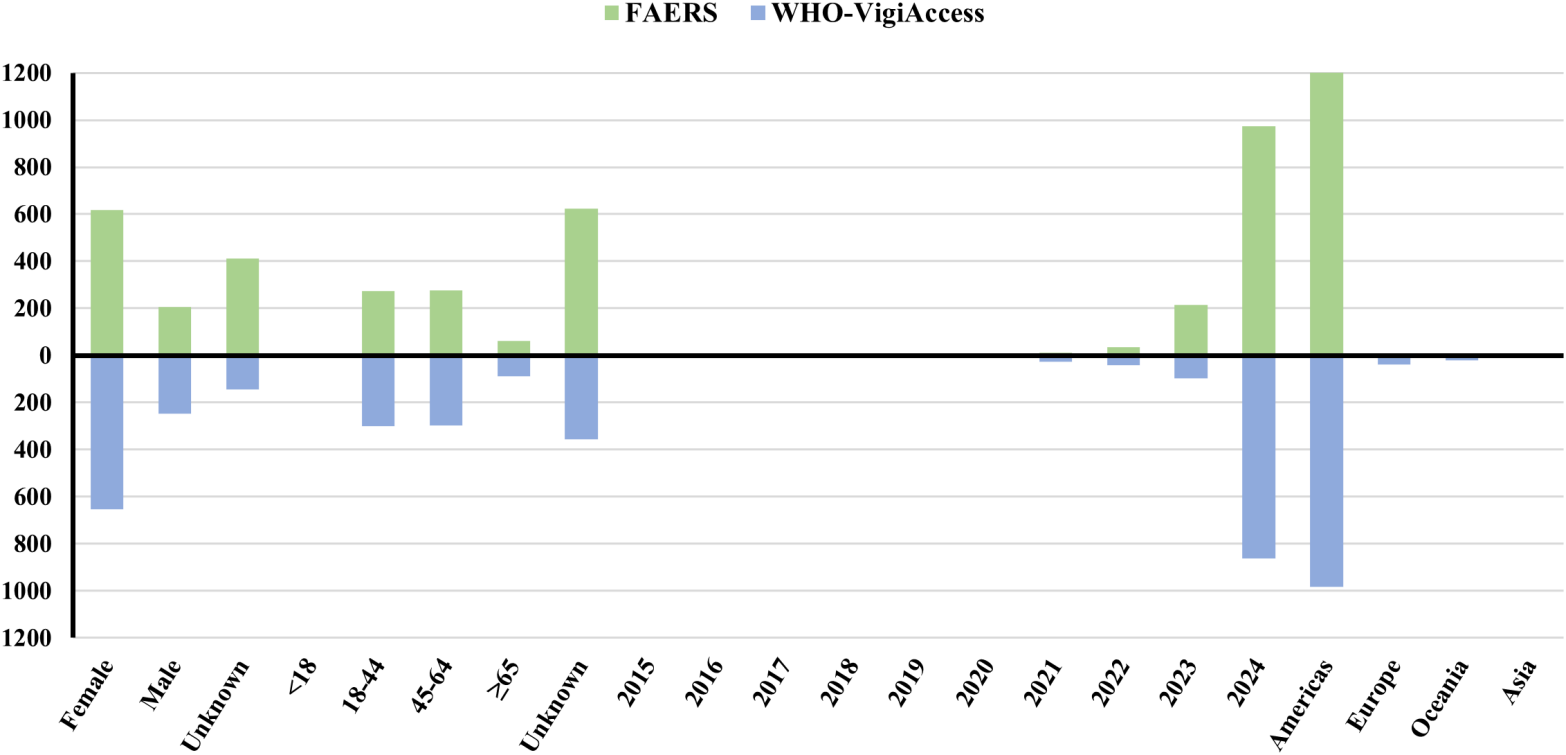
Characteristic analysis of AE reports in two databases.

The AE reports from both databases share the following characteristics (**Figure 2**): Regarding gender distribution, after excluding reports with unspecified data, adverse event reports were significantly more frequent in females than males, with male-to-female ratios of 3.02:1 (FAERS) and 2.65:1 (VigiAccess). Geographically, the majority of reports originated from the Americas, with minimal contributions from Asia. Nearly half of the reports lacked patient age information; among those with documented ages, 18-64-year-old patients accounted for 50.29%. Notably, AE reports associated with ublituximab in both databases have exhibited a marked surge in recent years. Temporally, reporting dates were predominantly clustered in 2024.

### 3.2 AE report analysis at SOC level

Injury, poisoning, and procedural complications, the largest SOC category in both databases (33.94% in FAERS and 31.91% in VigiAccess).

**Figure 3:**
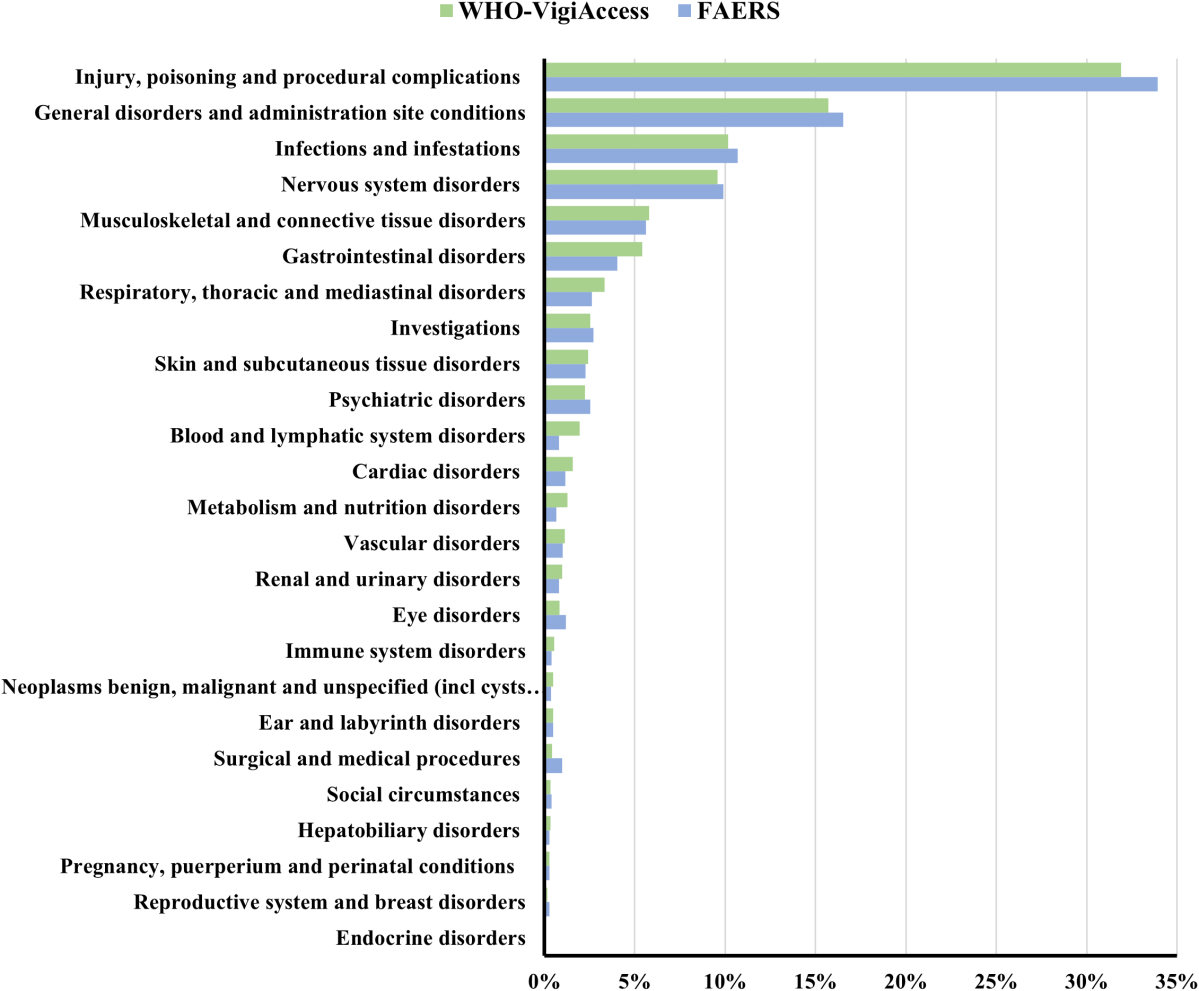
Proportion of AEs by SOC for ublituximab in two databases.

**Figure 3** exhibited the most robust signal strength in both databases, consistent with its known safety profile. General disorders and administration site conditions are the next most common. The following three SOC categories are infections and infestations, nervous system disorders, and musculoskeletal and connective tissue disorders.

### 3.3 AE report analysis at PT level

**Figure 4:**
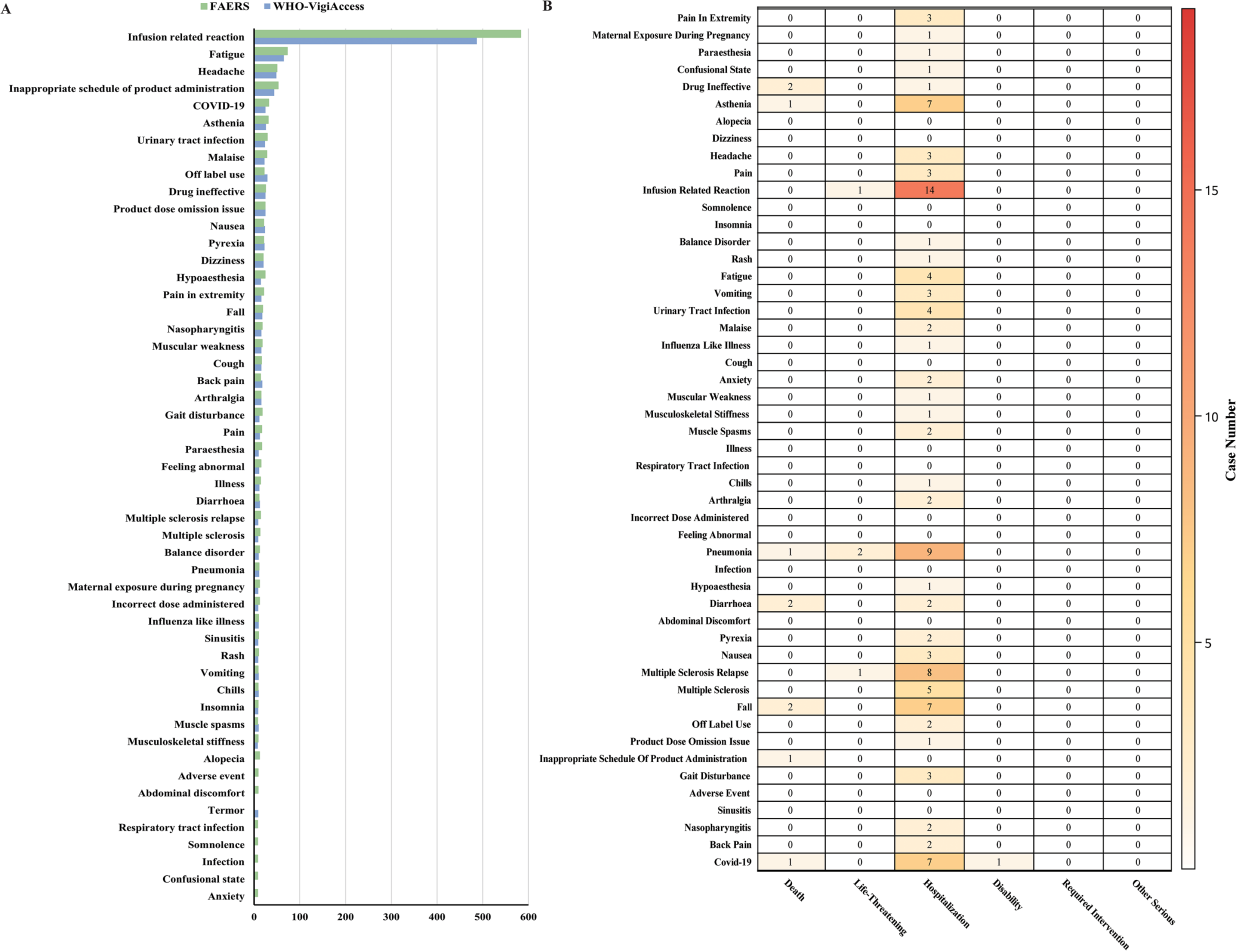
AE report analysis at PT level. **(A)** Top 50 PTs by frequency for AEs in two databases. **(B)** Outcomes of AEs by PTs.

Our study identified 52 PT-level positive signals associated with ublituximab through combined application of ROR and PRR, spanning 20 SOCs. Notably, four SOCs—cardiac disorders, vascular disorders, renal and urinary disorders, and social circumstances—were not documented in the drug label. **Figure 4** displays the top 50 PTs by frequency for AEs in both databases, along with AE outcomes by PT. The most frequent and strongest PTs across both databases were infusion-related reaction (IRR), accounting for 45.25% and 43.76% of top 30 PTs respectively—significantly exceeding other AEs. Common signals in both databases included infusion site discomfort, COVID-19 pneumonia, infusion site swelling, and maternal exposure during breastfeeding (**Tables 3**, **4**), etc. Unexpectedly, PTs such as hypoaesthesia, fall, gait disturbance, paraesthesia, balance disorder, alopecia, tremor, and somnolence, currently lack documentation in the drug label. 24 PT signals were detected by ROR, PRR, MHRA, BCPNN, and MGPS together. Among PTs resulting in death, drug ineffective, diarrhoea, and fall each had two cases.

**Table 3:** Signal strength of AEs at the PT level ranked by ROR in FAERS.

SOC	PT	Case reports	ROR (95% CI)	PRR (95% CI)	Chi Square	IC (IC025)	EBGM (EBGM05)
Injury, poisoning and procedural complications	Infusion related reaction	581	354.61 (322.53, 389.89)	263.10 (245.19, 282.32)	150249	8.02 (7.36)	260.33 (236.78)
General disorders and administration site conditions	Infusion site discomfort	3	88.81 (28.56, 276.14)	88.69 (28.57, 275.36)	259.19	6.47 (0.50)	88.38 (28.42)
Infections and infestations	COVID-19 pneumonia	8	18.51 (9.24, 37.06)	18.44 (9.23, 36.84)	131.91	4.20 (1.69)	18.43 (9.20)
General disorders and administration site conditions	Infusion site swelling	3	15.08 (4.86, 46.80)	15.06 (4.86, 46.67)	39.36	3.91 (0.29)	15.05 (4.85)
General disorders and administration site conditions	Infusion site erythema	4	15.05 (5.64, 40.14)	15.02 (5.64, 40.01)	52.33	3.91 (0.69)	15.01 (5.63)
General disorders and administration site conditions	Infusion site pain	6	13.85 (6.22, 30.88)	13.82 (6.21, 30.74)	71.33	3.79 (1.19)	13.81 (6.20)
Injury, poisoning and procedural complications	Maternal exposure during breast feeding	3	11.89 (3.83, 36.90)	11.87 (3.83, 36.80)	29.86	3.57 (0.23)	11.87 (3.82)
Nervous system disorders	Brain fog	3	9.94 (3.20, 30.87)	9.93 (3.21, 30.78)	24.09	3.31 (0.17)	9.93 (3.20)
Injury, poisoning and procedural complications	Intentional dose omission	7	9.14 (4.35, 19.20)	9.12 (4.35, 19.10)	50.58	3.19 (1.16)	9.11 (4.34)
Infections and infestations	Respiratory tract infection	8	8.85 (4.42, 17.72)	8.82 (4.42, 17.62)	55.50	3.14 (1.27)	8.82 (4.41)
Infections and infestations	Oral herpes	6	8.71 (3.91, 19.41)	8.69 (3.91, 19.32)	40.83	3.12 (0.96)	8.69 (3.90)
Nervous system disorders	Multiple sclerosis	13	7.24 (4.20, 12.49)	7.20 (4.19, 12.38)	69.46	2.85 (1.54)	7.20 (4.17)
Injury, poisoning and procedural complications	Inappropriate schedule of product administration	53	6.34 (4.83, 8.32)	6.21 (4.76, 8.10)	232.55	2.63 (2.10)	6.21 (4.73)
Respiratory, thoracic and mediastinal disorders	Acute respiratory failure	4	5.88 (2.20, 15.68)	5.87 (2.20, 15.63)	16.16	2.55 (0.28)	5.87 (2.20)
Nervous system disorders	Multiple sclerosis relapse	14	5.30 (3.13, 8.97)	5.27 (3.13, 8.89)	48.53	2.40 (1.29)	5.27 (3.12)
Infections and infestations	COVID-19	32	4.96 (3.50, 7.03)	4.90 (3.47, 6.91)	99.59	2.29 (1.62)	4.90 (3.46)
Infections and infestations	Urinary tract infection	29	4.78 (3.31, 6.89)	4.73 (3.29, 6.79)	85.42	2.24 (1.54)	4.73 (3.28)
General disorders and administration site conditions	Illness	14	4.69 (2.77, 7.93)	4.67 (2.77, 7.86)	40.36	2.22 (1.16)	4.66 (2.76)
Nervous system disorders	Hypoaesthesia	24	4.36 (2.91, 6.52)	4.32 (2.90, 6.43)	61.41	2.11 (1.35)	4.32 (2.89)
Musculoskeletal and connective tissue disorders	Muscular weakness	18	4.36 (2.74, 6.93)	4.33 (2.73, 6.86)	46.16	2.11 (1.22)	4.33 (2.72)
Infections and infestations	Upper respiratory tract infection	7	4.22 (2.01, 8.86)	4.21 (2.01, 8.81)	17.12	2.07 (0.56)	4.21 (2.00)
Injury, poisoning and procedural complications	Maternal exposure during pregnancy	12	4.08 (2.31, 7.19)	4.06 (2.31, 7.14)	27.72	2.02 (0.91)	4.06 (2.30)
Nervous system disorders	Balance disorder	12	3.77 (2.14, 6.65)	3.76 (2.14, 6.60)	24.30	1.91 (0.83)	3.76 (2.13)
General disorders and administration site conditions	Fatigue	73	2.66 (2.11, 3.36)	2.61 (2.08, 3.27)	73.25	1.38 (1.01)	2.61 (2.06)

**Table 4:** Signal strength of AEs at the PT level ranked by ROR in VigiAccess.

SOC	PT	Case reports	ROR (95% CI)	PRR (95% CI)	Chi Square	IC (IC025)	EBGM (EBGM05)
Injury, poisoning and procedural complications	Infusion related reaction	487	566.79 (511.77, 627.72)	431.52 (399.20, 466.46)	207746	8.74 (7.69)	428.34 (386.76)
Infections and infestations	COVID-19 pneumonia	10	27.06 (14.54, 50.38)	26.93 (14.51, 49.99)	249.62	4.75 (2.13)	26.92 (14.46)
General disorders and administration site conditions	Infusion site erythema	3	17.17 (5.53, 53.30)	17.15 (5.53, 53.14)	45.61	4.10 (0.32)	17.14 (5.52)
Injury, poisoning and procedural complications	Intentional dose omission	6	16.92 (7.59, 37.72)	16.88 (7.59, 37.53)	89.61	4.08 (1.28)	16.87 (7.57)
Metabolism and nutrition disorders	Tumour lysis syndrome	3	15.79 (5.09, 49.02)	15.77 (5.09, 48.87)	41.50	3.98 (0.30)	15.77 (5.08)
Injury, poisoning and procedural complications	Maternal exposure during breast feeding	3	13.14 (4.23, 40.78)	13.12 (4.23, 40.66)	33.59	3.71 (0.26)	13.12 (4.23)
General disorders and administration site conditions	Infusion site pain	4	12.74 (4.78, 33.99)	12.72 (4.78, 33.86)	43.19	3.67 (0.64)	12.72 (4.77)
Injury, poisoning and procedural complications	Inappropriate schedule of product administration	44	9.07 (6.73, 12.23)	8.90 (6.64, 11.92)	309.07	3.15 (2.48)	8.89 (6.60)
Respiratory, thoracic and mediastinal disorders	Acute respiratory failure	4	8.82 (3.31, 23.53)	8.81 (3.31, 23.45)	27.68	3.14 (0.49)	8.81 (3.30)
Infections and infestations	Respiratory tract infection	4	8.27 (3.10, 22.05)	8.25 (3.10, 21.97)	25.50	3.04 (0.46)	8.25 (3.09)
Infections and infestations	Upper respiratory tract infection	8	7.52 (3.75, 15.05)	7.49 (3.75, 14.96)	45.03	2.91 (1.16)	7.49 (3.74)
Infections and infestations	Urinary tract infection	24	7.33 (4.90, 10.96)	7.25 (4.87, 10.79)	129.55	2.86 (1.96)	7.25 (4.85)
Nervous system disorders	Multiple sclerosis	7	6.39 (3.04, 13.43)	6.37 (3.04, 13.35)	31.73	2.67 (0.91)	6.37 (3.03)
General disorders and administration site conditions	Therapeutic response unexpected	7	6.06 (2.89, 12.74)	6.05 (2.89, 12.67)	29.50	2.60 (0.87)	6.05 (2.88)
Nervous system disorders	Multiple sclerosis relapse	9	5.83 (3.03, 11.21)	5.81 (3.02, 11.14)	35.82	2.54 (1.06)	5.80 (3.02)
Respiratory, thoracic and mediastinal disorders	Pneumonitis	4	5.71 (2.14, 15.23)	5.70 (2.14, 15.18)	15.51	2.51 (0.26)	5.70 (2.14)
General disorders and administration site conditions	Illness	12	5.68 (3.22, 10.02)	5.65 (3.21, 9.94)	45.99	2.50 (1.25)	5.65 (3.20)
Injury, poisoning and procedural complications	Maternal exposure during pregnancy	9	5.36 (2.78, 10.31)	5.34 (2.78, 10.25)	31.76	2.42 (0.98)	5.34 (2.77)
Infections and infestations	Bronchitis	8	5.13 (2.56, 10.27)	5.11 (2.56, 10.21)	26.49	2.35 (0.85)	5.11 (2.55)
Infections and infestations	Sinusitis	9	4.86 (2.52, 9.35)	4.84 (2.52, 9.29)	27.45	2.28 (0.89)	4.84 (2.51)
Nervous system disorders	Balance disorder	10	4.66 (2.51, 8.68)	4.65 (2.50, 8.62)	28.63	2.22 (0.93)	4.64 (2.50)
Musculoskeletal and connective tissue disorders	Muscular weakness	16	4.54 (2.78, 7.43)	4.52 (2.77, 7.36)	43.86	2.17 (1.20)	4.51 (2.76)
Infections and infestations	Nasopharyngitis	16	3.82 (2.34, 6.26)	3.80 (2.33, 6.20)	33.12	1.93 (1.00)	3.80 (2.32)
Injury, poisoning and procedural complications	Product dose omission issue	25	3.12 (2.10, 4.62)	3.09 (2.09, 4.56)	35.48	1.63 (0.95)	3.09 (2.08)

**Figure 5:**
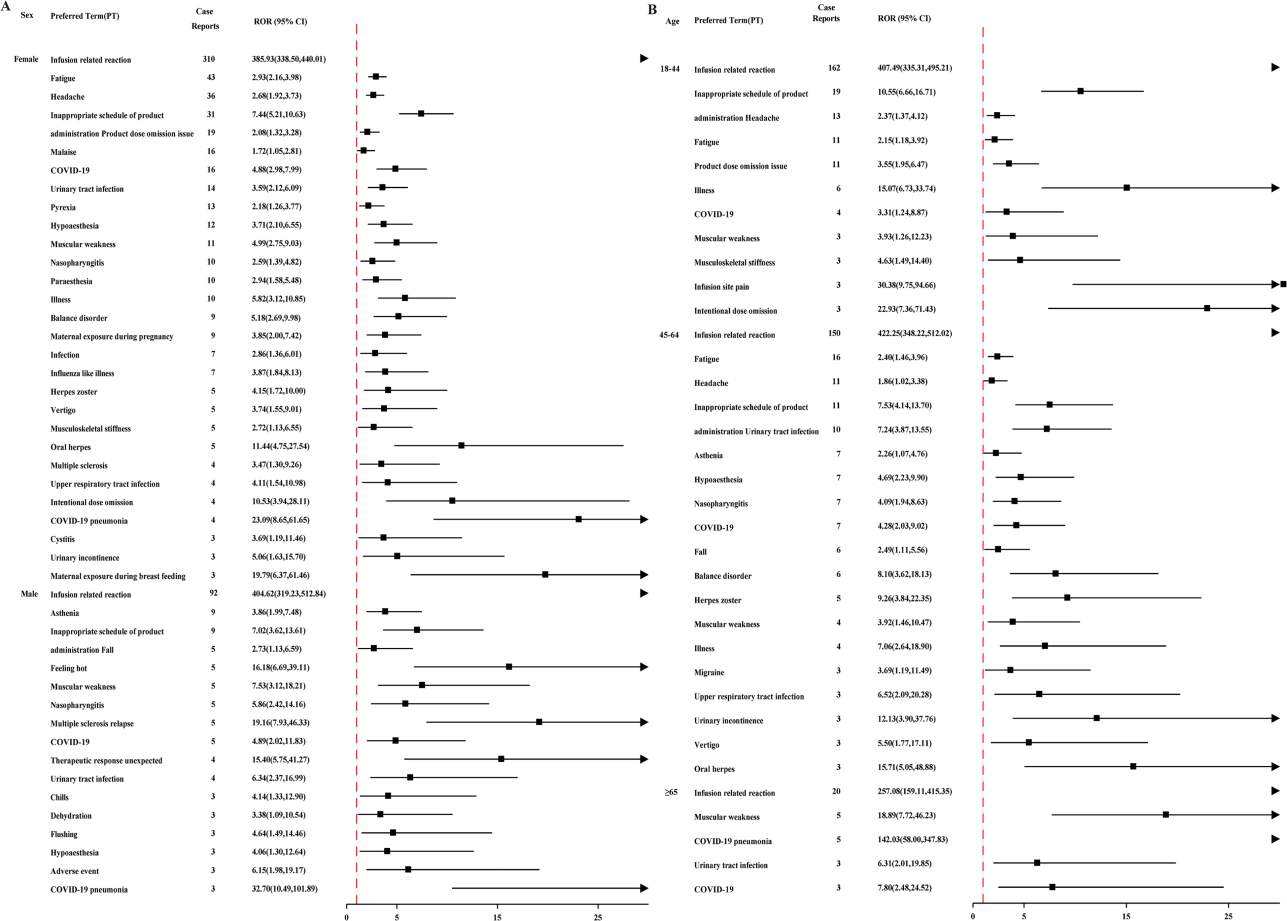
Stratified analysis of AEs by gender and age in FAERS. **(A)** Top 50 PTs ranked by frequency of positive signals across different genders. **(B)** Top 25 PTs ranked by frequency of positive signals across different age.

A pronounced sex- and age- related disparities in AE occurrence is evident across both databases (**Figure 5**). Ublituximab is associated with a significantly elevated risk of urinary tract infection (UTI), IRR, COVID-19 pneumonia, and muscular weakness were reported disproportionately more frequently in male patients receiving ublituximab. In female patients, headache, fatigue, cystitis, and maternal exposure during both pregnancy and breastfeeding showed disproportionality signals (**Figure 5A**). IRR reports were more frequent in younger cohorts (18–44 years) compared to older groups (45–64 and ≥65 years). Headache incidence was elevated in the 18–44 age range, whereas muscular weakness and COVID-19 predominated in patients aged ≥65 (**Figure 5B**).

### 3.4 Time to event onset

**Figure 6:**
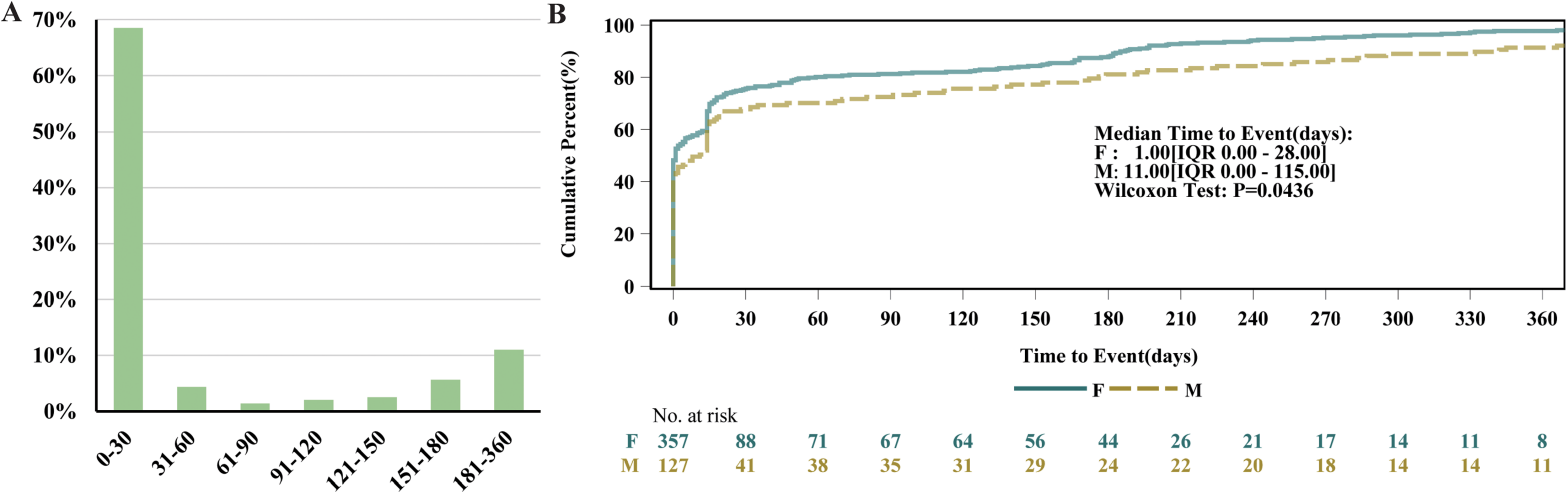
Temporal dynamics of adverse events in FAERS. **(A)** Time to event report distribution of AE reports. **(B)** Cumulative incidence of AEs by sex.

Excluding reports with unknown timing (42.11%), the majority of AEs occurred within 0–30 days (39.68%) (**Figure 6A**). The median time to AE onset for ublituximab was 5 days, indicating that half of the patients experienced AEs within 5 days of administration. further shows that women receiving ublituximab are more prone to early AEs (**Figure 6B**).

### 3.5 Drug-drug interaction

**Figure 7:**
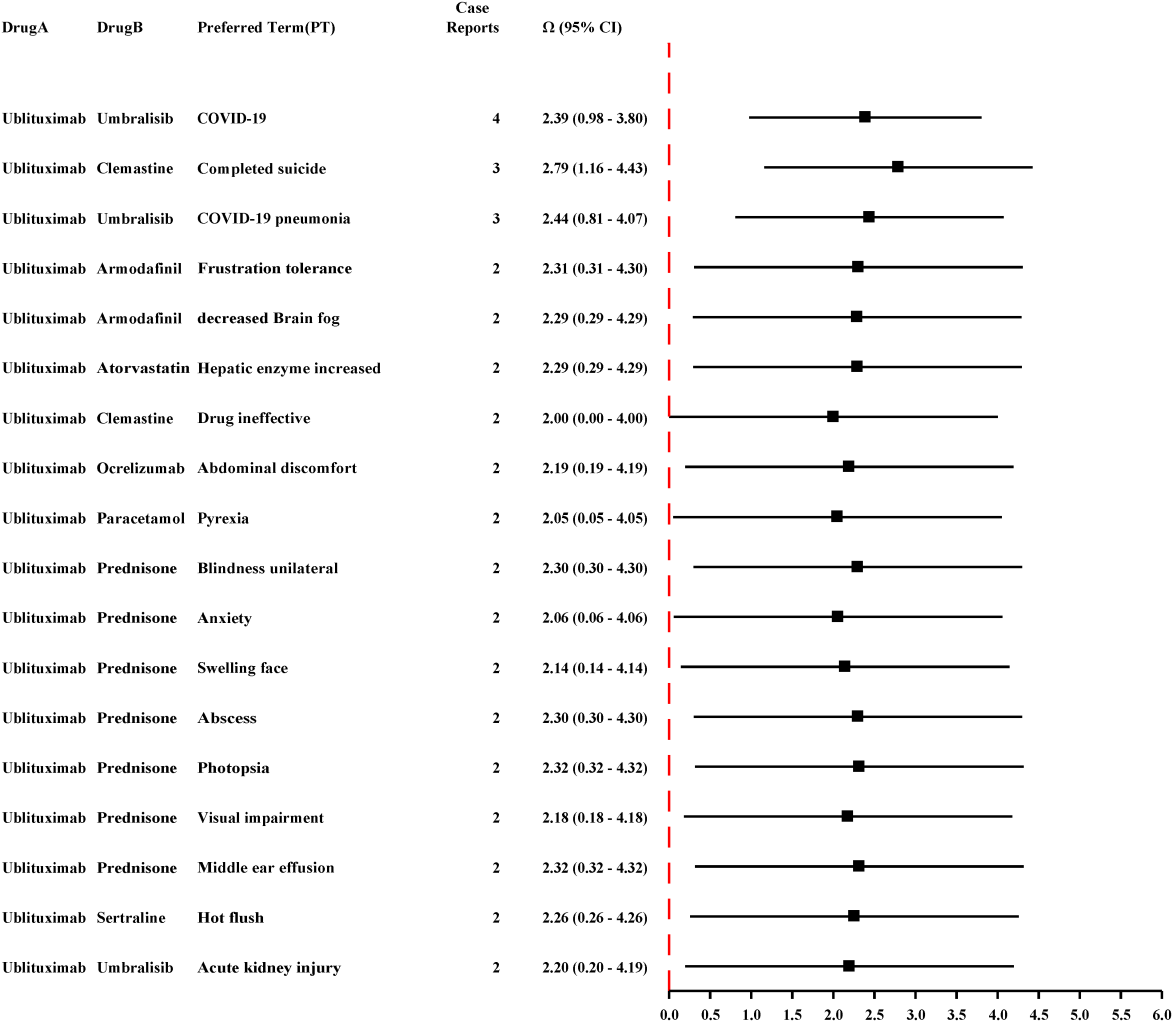
PTs associated with drug-drug interactions among the top 50 PTs.

Our analysis of the FAERS reveals that ublituximab is frequently co-administered with umbralisib, clemastine, armodafinil, atorvastatin, ocrelizumab, paracetamol, prednisone, and sertraline (**Figure 7**). A disproportionality signal for completed suicide was observed in patients co-administered ublituximab and clemastine, while ublituximab use with corticosteroids (e.g., prednisone) was associated with disproportionality signals for pyrexia, unilateral blindness, anxiety, facial swelling, abscess, photopsia, visual impairment, and middle ear effusion, which may be related to exacerbated metabolic disorders and deepened immunosuppression.

## 4 Discussion

In December 2022, ublituximab received its first global approval in the United States for treating adult patients with MS (14), encompassing clinically isolated syndrome, relapsing-remitting MS, and active secondary-progressive MS (5). Rigorous clinical trials have established its efficacy and safety profile. However, comprehensive post-marketing studies on long-term safety and pharmacovigilance remain limited.

The geographical distribution of AE reports exhibits marked heterogeneity, with a disproportionate majority of cases in both databases originating from the Americas. This pattern aligns with global disparities in MS prevalence, which ranges from 290 per 100, 000 in Canada—the highest reported rate—to 8.6 per 100, 000 in Southeast Asia, the lowest observed globally (3). Regulatory approval status further exacerbates reporting biases: While ublituximab is licensed in the United States, Canada, and Europe, it remains unapproved in Asia and Oceania, regions that consequently contribute minimal AE data. The substantial proportion of reports with unspecified gender and age in pharmacovigilance datasets reveals limitations in real-world drug safety surveillance. Integrating electronic health records with pharmacovigilance databases would mitigate data quality issues arising from missing demographic information and enhance AE signal detection reliability.

Consistent with core AE signals from database analysis, Phase 2/3 trials confirmed infusion-related reactions (IRR) and infections as the most common AEs of ublituximab (incidence ≥10%) (7, 14). In Phase 3 ULTIMATE trials, IRR affected 48% of patients, mainly presenting as fever (28%), chills (24%), headache (22%) and infusion site reactions (18%)—consistent with real-world IRR manifestations (FAERS: ROR 354.61, 95% CI 322.53-389.89) (14). This 48% IRR incidence exceeded other anti-CD20 monoclonals (ofatumumab: 20%; ocrelizumab: 34%), aligning with real-world observations of IRR as the strongest AE signal. For infections, trials reported upper respiratory tract infections (15%), urinary tract infections (8%), and oral herpes (4%) in ublituximab-treated patients, all validated by real-world data with significant disproportionality (FAERS ROR: 4.22, 4.78, 8.71 respectively). Neurological AEs partially overlapped between trials and real-world settings. Trials reported headache (17%), fatigue (14%), and tremor (3%) (9), while real-world data identified headache (female-predominant), fatigue (FAERS: ROR 2.66, 95% CI 2.11-3.36), and tremor as unlabeled signals. Novel signals (hypoaesthesia, balance disorder, brain fog; FAERS ROR: 4.36, 3.77, 9.94 respectively) were unprominent in trials, possibly due to exclusion criteria or shorter follow-up (trials: 96 weeks; real-world: up to 9 years) (7, 15). Reproductive toxicity signals (FAERS ROR: pregnancy 4.08; breastfeeding 11.89) matched trial warnings about fetal B-cell depletion risk (16, 17), though trials lacked long-term neonatal outcome data, underscoring real-world surveillance value (18).

The sex-based disparity in AE occurrence reflects the female predominance consistently observed in MS, with a 2–3-fold higher incidence in women than men (1). And the convergence of sex-specific disease susceptibility and AE reporting trends underscores the need for gender-stratified pharmacovigilance analyses in post-marketing surveillance. For instance, headache is more common in women. Though statistically significant, headache is a common non-serious AE of ublituximab, and healthcare professionals are advised to monitor headache frequency in female patients, adjusting the dose only if necessary. Moreover, urinary tract infection (UTI), infusion-related reaction (IRR), COVID-19 pneumonia, and muscular weakness were reported significantly more frequently in male patients treated with ublituximab, all of which exhibited a disproportionality signal. In contrast, fatigue, cystitis, and maternal exposure during pregnancy or breastfeeding were associated with a disproportionality signal in female patients receiving the same treatment. This observation may be related to the different immune - regulatory effects of estrogen and testosterone. Women receiving ublituximab are more prone to early AEs. Women may exhibit reduced CYP450 enzyme activity and higher body fat levels, which could potentially prolong the pharmacokinetics of monoclonal antibodies such as ublituximab and contribute to more frequent reports of early AEs. We suggest clinicians maintain heightened vigilance during initial ublituximab treatment in female patients.

Injury, poisoning and procedural complications is the most frequent SOC in both databases, with IRR (ROR 354.61, 95% CI 322.53-389.89)—characterized by fever, nausea, chills, vomiting, headache, rash, and bronchospasm, etc (19)—being the most frequent and strongly associated AE. Although most IRRs are generally mild to moderate (20), severe reactions can be life-threatening and are the most common cause of hospitalization. Ublituximab’s CD20-binding mechanism and Antibody-Dependent Cell-mediated Cytotoxicity -enhanced B-cell depletion may be associated with IRR signal strength, with rapid infusion potentially contributing. Clinical trials have indicated a higher incidence of IRR related systemic reactions with ublituximab (48%) than with ofatumumab (20%) and ocrelizumab (34%) (21). Ublituximab administration requires medical supervision, including patient education and monitoring before and during treatment. The high frequency of IRR signals suggests that further prospective studies are needed to evaluate the efficacy of premedication strategies in clinical practice. We suggest titrating infusion duration and rate based on real-time monitoring of blood pressure, respiratory rate, and oxygen saturation to optimize tolerance.

While current evidence lacks consensus on age-related susceptibility to IRRs, our analysis reveals a notable disparity: heightened IRR vulnerability in younger populations receiving ublituximab. Mechanistically, the higher frequency of IRR reports in patients under 65 years may be related to more robust immune effector functions—particularly enhanced NK cell activity and complement system activation—that amplify cytokine release dynamics upon pharmacological stimulation. Therapeutic conservatism in elderly care—including routine dose reductions and prolonged infusion durations—likely contributes to their reduced IRR manifestation. Notably, the strong signal association with inappropriate administration schedules (ROR 6.34, 95% CI 4.83-8.32) highlights the need for rigorous adherence protocols. Suboptimal dosing intervals may induce anti-drug antibody formation (22), compromising efficacy and safety, particularly in outpatient settings where ublituximab’s narrow therapeutic window demands precise pharmacokinetic management.

Moreover, ublituximab has demonstrated a significantly elevated risk of maternal exposure during both pregnancy and breastfeeding, highlighting deficiencies in the current clinical management of drug-related reproductive toxicity. As an anti-CD20 monoclonal antibody, ublituximab can cross the placental barrier, potentially impairing fetal B-cell development (16). CD20 plays a pivotal role in B-cell maturation (23), and its depletion may lead to neonatal hypogammaglobulinemia. Although monoclonal antibodies, with their large molecular weight (150 kDa), are typically excreted in breast milk at low levels (<1% of maternal serum concentration), prolonged breastfeeding may still expose infants to immunosuppressive risks. Therefore, we recommend that clinicians confirm the pregnancy therapy. The disproportionality signal of maternal exposure during pregnancy and breastfeeding warrants clinical attention, and additional controlled studies are required to clarify the reproductive safety profile of ublituximab. For instance, patients using ocrelizumab should avoid pregnancy for 6–12 months post-discontinuation (17). Given ublituximab’s half-life of 22 days, clinicians should provide comprehensive guidance on adjusting pregnancy and breastfeeding timelines based on drug clearance rates. The strong signal of intentional dose omission (ROR 6.34, 95% CI 4.83-8.32) in FAERS reflects the potential risk of treatment discontinuation associated with ublituximab in clinical use. We should encourage healthcare providers to report the specific reasons for dose omissions to facilitate further analysis and the development of targeted interventions.

General disorders and administration site conditions is the SOC with the most positive signals in FAERS. Fatigue, infusion site erythema, infusion site pain, and illness are AEs shared between the two databases. A highly significant positive signal was detected in FAERS—infusion site discomfort (ROR 88.81, 95% CI 28.56-276.14). Ublituximab can activate immune cells through complement activation and cytokine release, leading to vasodilation and the release of inflammatory mediators, which could contribute to infusion site erythema. It may also induce localized burning, swelling, and tightness, causing infusion site discomfort. Furthermore, this process releases a significant amount of inflammatory mediators (24), which activate local nociceptive neurons, resulting in infusion site pain. Mechanistically, the IgG1 subtype of ublituximab activates the complement cascade, generating anaphylatoxins C3a and C5a, which stimulate mast cells to release histamine, increasing vascular permeability and causing localized edema and compressive discomfort. However, whether this mechanism leads to significant allergic reactions in clinical practice requires further research and monitoring. Additionally, the membrane attack complex directly damages vascular endothelium, contributing to ischemic tissue pain (25). These local reactions may be linked to systemic infusion reactions and require comprehensive management. Although the actual number of reported cases for these signals is limited (e.g., only 3 cases of infusion site discomfort), the high ROR values suggest the need for validation with larger sample sizes in future studies. We observed that fatigue (ROR 2.66, 95% CI 2.11-3.36 in FAERS) shows high frequency but weak signal strength, potentially reflecting multifactorial origins including disease-related symptoms, concomitant immunomodulator effects, and subjective reporting variability. This underscores the need for standardized fatigue assessment (e.g., FACIT-F) and integrated management approaches combining pharmacological and non-pharmacological interventions in moderate-to-severe cases.

Infections and infestations are a significant cause of death and hospitalization. Common AEs in this category include respiratory tract infection, oral herpes, upper respiratory tract infection, urinary tract infection, bronchitis, sinusitis, and nasopharyngitis. Recent studies suggest that the rapid B-cell depletion kinetics of ublituximab may exacerbate early infection risks (7).B cells play a pivotal role in humoral immunity, contributing to antibody production and the secretion of cytokines such as IL-6 and TNF-α (26). IL-10 secreted by B cells can further modulate immune responses by inhibiting TNF-α release from macrophages (27). Ublituximab treatment has been found to increase the risk of neutropenia, a known risk factor for severe infections (18). Patients with MS often exhibit immune dysregulation or organ dysfunction, and B-cell depletion further heightens their susceptibility to infections. Among these AEs, COVID-19 pneumonia (ROR 18.51, 95% CI 9.24 -37.06) demonstrates the strongest association and highest frequency. Anti-CD20 monoclonal antibody therapy may delay clearance of the COVID-19 virus and increase severe illness risk (18). When developing treatment plans, clinicians should assess individual risk factors including comorbidities, age, and immune status. Continuous monitoring of respiratory symptoms and pulmonary imaging changes during treatment is essential for prompt identification of COVID-19 pneumonia. While pandemic-era data reporting may reflect surveillance bias, this signal warrants attention. We recommend establishing ≥2-year follow-up cohorts to monitor long-term sequelae for robust real-world evidence.

Respiratory tract infection (ROR 8.85, 95% CI 4.42-17.72) and oral herpes (ROR 8.71, 95% CI 3.91-19.41) also demonstrate strong associations with ublituximab. Current evidence indicates that known anti-CD20 monoclonal antibodies, such as rituximab and ocrelizumab, are associated with an increased risk of respiratory tract infections, a mechanism attributed to B-cell depletion (15). We propose that chronic depletion reduces serum IgG/IgM levels, with low IgG levels being closely associated with recurrent respiratory infections (28). The association between ublituximab-induced B-cell depletion and increased infection risk highlights the need for further prospective controlled studies to evaluate the efficacy of preventive interventions, such as pneumococcal, influenza, or COVID-19 vaccinations administered ≥4 weeks before treatment initiation. For patients with recurrent infections or IgG <5 g/L, long-term low-dose azithromycin may mitigate pneumonia risk. Oral herpes highlights the need for clinicians to implement preventive antiviral therapy and mucosal immune monitoring in patients.

Additionally, a positive signal for urinary tract infection (UTI) (ROR 4.78, 95% CI 3.31-6.89 in FAERS) has been identified, with a high frequency of occurrence across both databases. We observed that men exhibit greater ublituximab-associated UTI risk than women, despite women’s higher physiological baseline risk. We hypothesize this may reflect women’s more robust immune systems, which, while increasing autoimmune susceptibility, enhance pathogen defense. We recommend that prioritizing urinary management strategies, including intermittent catheterization and prophylactic antibiotics, in male ublituximab recipients, with urinary IgA monitoring to inform IVIG thresholds. Future studies should elucidate sex hormone-mediated immune regulation mechanisms underlying UTI susceptibility.

Within the nervous system disorders category, we identified a positive signal for brain fog (ROR 9.94, 95% CI 3.20-30.87) in FAERS, which exhibited the strongest signal intensity among this SOC. Monoclonal antibodies’ molecular size restricts BBB penetration (29), though pathological conditions like MS may increase BBB permeability (30). We hypothesize CNS access could modulate neuroinflammatory pathways, potentially inducing cognitive dysfunction. Additionally, FAERS data revealed strong signals for MS (ROR 7.24, 95% CI 4.20-12.49) and MS relapse (ROR 5.30, 95% CI 3.13-8.97), suggesting incomplete disease suppression in some patients. While phase 3 ULTIMATE I and II trials demonstrated significant relapse rate reduction (9). However, treatment efficacy may be influenced by dosage, administration frequency, adherence, and MS’s phenotypic and immune response heterogeneity. Future research should further explore the efficacy of ublituximab across different MS subtypes and optimize treatment strategies to minimize relapse risks. Hypoaesthesia (ROR 4.36, 95% CI 2.91-6.52 in FAERS) and balance disorder (ROR 3.77, 95% CI 2.14-6.65 in FAERS) are among the new safety signals we have identified, which have yet to be included in the drug labeling. It is important to acknowledge that these neurological signals cannot be clearly attributed to ublituximab alone, as patients with MS inherently exhibit increased BBB permeability, abnormal central immune surveillance, and ongoing disease progression that may contribute to such symptoms (31). While B-cell depletion might potentially modulate the neuroimmune microenvironment, the observed signals may also reflect underlying MS pathology or disease evolution. Although these AEs are not currently highlighted in clinical practice, their frequent reporting in real-world settings warrants further investigation to clarify their underlying causes and clinical significance for comprehensive patient safety assurance.

In terms of the respiratory, thoracic, and mediastinal disorders category, acute respiratory failure (ROR 8.82, 95% CI 3.31-23.53 in VigiAccess) is rare but exhibits a strong signal. While respiratory infections were commonly reported AEs in ublituximab clinical trials, acute respiratory failure was not specifically highlighted. However, we observed significant acute respiratory failure associations in real-world settings, warranting vigilance and further investigation into its mechanisms and risk factors. We recommend pretherapeutic screening for acute respiratory failure incorporating lung function (DLCO) quantification, high-resolution CT imaging, and anti-AChR antibody profiling. During treatment, dynamic biomarker tracking may optimize risk mitigation through adaptive therapeutic modulation.

Regarding metabolism and nutrition disorders in the VigiAccess database, tumour lysis syndrome (TLS) (ROR 15.79, 95% CI 5.09-49.02) is rare but demonstrates a strong association. Though absent from clinical trials, real-world disproportionality signals suggest this rare AE may stem from rapid lytic B-cell clearance, culminating in systemic release of intracellular potassium, phosphate, and nucleic acid derivatives - a pathogenic pathway requiring biochemical validation. Therefore, in clinical practice, TLS is a rare but strongly associated adverse event with ublituximab, which requires clinical awareness in high-risk patients. The potential value of electrolyte and renal function monitoring, or the use of uric acid-lowering agents (e.g., allopurinol, rasburicase), for TLS prevention warrants prospective controlled investigation.

We have identified additional positive signals associated with ublituximab in both databases. Currently, signals not yet included in the drug label include hypoaesthesia, fall, gait disturbance, paraesthesia, balance disorder, alopecia, tremor, and somnolence. These positive signals span multiple SOCs, warranting clinical attention and further validation. These findings highlight critical safety considerations that may merit additional regulatory review to determine their suitability for potential drug label updates.

Ublituximab’s AEs exhibited a pronounced early-onset pattern, followed by a gradual decline. Acute reactions manifest as immediate-onset events (e.g., IRR), primarily occurring during initial infusion. This reflects the drug’s rapid immune-mediated mechanism (32). While mid-term events correlate with B cell depletion-induced cumulative toxicity or secondary immunodeficiency. Late-onset risk attenuation suggests immune adaptation or target cell clearance completion. Clinicians must closely monitor vital signs, allergic reaction markers, and inflammatory indicators during the first infusion, and ensure regular follow-up and periodic assessments for long-term therapy patients.

Our study reveals that ublituximab-based combination therapies require urgent mechanistic investigation given their complex immunomodulatory profiles. The strongest suicide disproportionality signal was observed in the ublituximab-clemastine combination, which may be related to clemastine’s anticholinergic effects exacerbating cognitive deficits or depression in MS patients (33). Given that MS itself is associated with increased risks of cognitive impairment and psychiatric symptoms, these observations may reflect underlying disease pathology rather than a direct or indirect effect of ublituximab. While ublituximab-mediated B-cell depletion could theoretically modulate neuroinflammatory cytokines, the contribution of the drug to these psychiatric outcomes remains unclear and requires further validation. Of course, this signal is only a preliminary finding and cannot be used as a basis for clinical medication restrictions. In clinical practice, it is necessary to pay more attention to observing the mental state of patients taking the combination therapy, especially when psychotropic drugs are used simultaneously. Dual B-/T-cell immunosuppression emerges as a critical concern, particularly in ublituximab-umbralisib combinations. This regimen significantly amplifies opportunistic infection risks due to humoral immunity suppression and PI3Kδ-mediated T-cell inhibition. For patients receiving these two drugs in combination, we recommend that clinicians regularly monitor viral loads and lymphocyte subsets and initiate antiviral therapy early. For those with severe symptoms, clinicians should consider suspending the use of immunosuppressive agents. Additionally, when ublituximab is used in combination with corticosteroids (e.g., prednisone), it may exacerbate metabolic disorders and deepen immunosuppression, increasing the risk of diseases such as pyrexia, unilateral blindness, anxiety, facial swelling, abscess, photopsia, visual impairment, and middle ear effusion. Increased hepatic enzymes, hot flushes, abdominal discomfort, and drug ineffectiveness are also AEs associated with the combination of ublituximab and other drugs. Combination therapy potentially amplifies ublituximab’s immunosuppressive effects, suppressing T cell function and elevating infection risks. Infections and CNS-related disorders emerge as primary risks in ublituximab-containing polypharmacy. Systematic investigation of ublituximab-based combinations is required to optimize therapeutic efficacy while mitigating adverse effects.

## 5 Limitations

While our pharmacovigilance analysis revealed critical safety signals for ublituximab, inherent methodological constraints of spontaneous reporting systems must be acknowledged. FAERS and VigiAccess data contain systemic biases including underreporting, duplicate entries, and incomplete documentation (e.g., missing patient demographics, dosing parameters, or AE severity grading). Furthermore, the neurological adverse event signals identified in this study may be influenced by confounding factors. These symptoms may either be related to ublituximab or reflect the underlying pathological features or disease progression of MS. Due to the lack of relevant clinical details in the database, it is currently impossible to clearly attribute them to ublituximab. These limitations preclude causal inference, as temporal associations in spontaneous reports only indicate statistical probability rather than validated drug-event relationships. Additionally, managing AEs of immunosuppressive drugs is indeed highly challenging, closely related to the complex pharmacological properties of the drugs, individual patient differences, and the highly dynamic balance requirements of the immune system.

## 6 Conclusion

This study confirmed ublituximab’s common AEs and identified potential AE signals. These findings highlight the need for further regulatory review to assess whether the identified signals justify updates to drug labeling and therapeutic guidelines. Through comprehensive safety evaluation across diverse populations and identification of high-risk drug-drug interactions, we achieved personalized pharmacovigilance. Data mining from FAERS and VigiAccess databases offered essential reference information for clinicians, regulatory agencies, and patients. While most AEs are mild and self-limiting, severe cases may lead to hospitalization or mortality. Healthcare professionals should particularly monitor signals not adequately addressed in drug labels. Future research should build upon these identified AE signals to establish clinical incidence rates and definitive causal relationships with ublituximab.

## Data Availability

The original contributions presented in the study are included in the article/supplementary material. Further inquiries can be directed to the corresponding author.
